# Commentary on “Reflections on the Inclusion of Physicians as Educators in Community Hospitals”

**DOI:** 10.1590/0100-6991e-20243864-en

**Published:** 2024-12-02

**Authors:** HERMILA TAVARES VILAR GUEDES

**Affiliations:** 1 - Universidade do Estado da Bahia - UNEB,, Professora Adjunta - Curso de Medicina - Departamento de Ciências da Vida - Salvador - BA - Brasil; 2 - Escola Bahiana de Medicina e Saúde, Professora Adjunta - Salvador - BA - Brasil

These considerations were prepared based on the invitation to reflect, contained in the text of this journal’s Letter to the Editors, as quoted above^1^.Thus, the objective is to broaden the approach and deepen the discussion of the various aspects contained therein. After all, reflection is never excessive and remains an essential human function. With the permission of the fellow authors of the “letter” and the editors, I would like to encourage readers to think critically.

While the text highlights important issues, such as the need for training in teaching and preceptorship-two distinct functions-the challenge of involving physicians in educational roles within the Brazilian context demands a deeper and more critical analysis.

In truth, the role of preceptors cannot be examined in isolation. It is crucial to underscore that, according to the Brazilian National Curricular Guidelines for medical courses (DCNM), medical education must be intrinsically linked to the Unified Health System (SUS), which, beyond its principles of universality, comprehensiveness, and equity, as outlined in Article 27, plays a pivotal role in training health professionals, serving as a practice environment across various levels of education, including postgraduate studies and research. Finally, while comparing the US and Brazilian health systems is a complex endeavor that requires careful consideration of structural and financial differences, this analysis lies outside the scope of this text.

Addressing the role of physicians as educators in health units requires a closer look at the “Service Preceptor” position as part of a broader strategy to strengthen the teaching-service relationship, which, in order to be effective, must be supported by adequate working conditions and robust pedagogical training.

The education of physicians in Brazil cannot ignore regional inequalities and the collective logic that underpins the SUS. Brazil faces an urgent need for general practitioners who are well-prepared to work in diverse environments-a diversity that extends beyond geographical and administrative settings. 

The demands of the SUS exceed technical competence, requiring professionals to tackle collective health challenges while providing coordinated, comprehensive care. The so-called Primary Care, to which comprehensiveness and coordination are pillars, requires broad technical skills. There is a common misconception that Primary Care deals only with the “simplest” aspects of medicine. However, in reality, medical professionals in this field require robust technical training from the outset, with strong clinical skills, to effectively serve as the foundational link in community healthcare. 

The significant differences between community hospitals in the U.S. and public hospitals in Brazil must also be acknowledged. In the U.S., community hospitals often enjoy greater financial autonomy and focus on serving local populations. By contrast, public hospitals in Brazil are central to providing care for vulnerable populations, many of which serve disproportionately large regions despite being underfunded and overburdened. This disparity limits the direct application of U.S. models to the Brazilian context.

A critical challenge lies in the lack of pedagogical training for preceptor physicians. However, responsibility for this gap can be traced to systemic issues within the educational and healthcare frameworks, where physicians are allowed to assume teaching roles without the necessary support or training-something implicitly allowed by Law 8080/1990. 

One issue raised in the referenced text that warrants further discussion is the terminal nature of undergraduate medical courses in Brazil, where medical residency is not mandatory. While Medical Residency Programs (MRPs) aim to produce specialists, they do not necessarily prepare physicians for preceptorship. However, some MRPs offer their graduates opportunities to continue as preceptors for a set period. These arrangements can serve as valuable internships, providing practical training for preceptorship and contributing to professional development.

Another pressing concern is the rapid expansion of medical schools and the increase in available spots in existing courses; this growth must be scrutinized carefully. Public institutions, many of which lack faculty (and proper incentives for them), resources, infrastructure, modern management practices, equipment, and supplies, should be prioritized. The justification often centers on insufficient funding. However, this expansion appears to cater to the interests of profit-driven institutions, whose commitment to delivering high-quality education often takes a backseat to financial gains derived from enrollment fees. This includes the public funds allocated to these institutions, which could potentially enhance both the quality and capacity of professional education in public courses.

It is also worth mentioning that the training of new physicians has not followed a logical distribution between regions since it is difficult to understand how to authorize the operation of a medical program in locations with clear healthcare problems, a deficit of units and even qualified physicians. The establishment of medical programs in these areas has often been driven by political and economic interests, overshadowing technical considerations for optimizing education. Many such programs adjust their subject offerings based on the sporadic availability of instructors from more developed regions, as these professionals often do not reside in the municipalities where they teach.

In fact, many physicians working in health units, both in urban centers and rural areas, also serve as preceptors or professors, often without prior training for these roles. Also, working in this field often reveals a talent for teaching, which can evolve into a fulfilling and impactful career in education and/or preceptorship. The issue arises when a lack of educator training is compounded by deficiencies in undergraduate education, exacerbated by the proliferation of poorly assessed courses. A skilled technician who cultivates pedagogical abilities can become an exceptional preceptor early on and may even pursue a teaching career.

The text highlights the burden on physicians serving as preceptors, attributing it to the precarious conditions of public health services but without providing an in-depth analysis. It is essential to recognize that the involvement of resident physicians and interns in healthcare services can significantly impact the quality of care-positively or negatively-depending on how it is managed. 

The collaboration between universities and hospitals needs to be reimagined to ensure medical education emphasizes real-world SUS environments, such as Basic Health Units (UBSs) and public hospitals. Interministerial Ordinance No. 1124/2015, which introduced the Organizational Contracts for Public Action in Education and Health (COAPES), was not widely embraced and has been inconsistently implemented in various contexts. There is a need to strengthen the alignment between education and service delivery, ensuring that medical training meets the demands of the SUS. COAPES management must be genuinely collaborative, involving both health and education leaders, with a primary focus on the technical quality of medical training and the care delivered to the community.

The gap between medical education and SUS requirements will persist until public health policies prioritize the establishment of robust career paths for all health professionals, not just physicians, with adequate working conditions and protected time for teaching and supervision.

While important, the proposed solutions-such as training for preceptorship and teaching roles, along with the development of teaching guides-remain inadequate in addressing the broader challenges of the Brazilian context.

The alignment of public healthcare policies with professional training must be seamless, focusing on technical competence as a foundation while clearly and transparently prioritizing the community and the individual as the central focus of health and education initiatives..
